# CXCL12 as a Potential Hub Gene for N-Acetylcysteine Treatment of T1DM Liver Disease

**DOI:** 10.3390/biom15020176

**Published:** 2025-01-25

**Authors:** Menglong Zhao, Mingzheng Han, Shuaihao Guo, Zhaoxin Tang

**Affiliations:** College of Veterinary Medicine, South China Agricultural University, Guangzhou 510642, China; zml1130@stu.scau.edu.cn (M.Z.); mz@stu.scau.edu.cn (M.H.); 1434760553@stu.scau.edu.cn (S.G.)

**Keywords:** T1DM liver disease, beagles, NAC, CXCL12

## Abstract

The etiology of type 1 diabetes mellitus (T1DM) is intricate, leading to its classification as an autoimmune metabolic disorder. T1DM often coexists with various visceral diseases. N-acetylcysteine (NAC) is widely acknowledged for its potent antioxidant properties. Studies have demonstrated that the combination of NAC and insulin can effectively alleviate iron-induced nephropathy in T1DM and mitigate oxidative stress injury in skeletal muscle associated with the condition. However, the potential impact of NAC alone on liver disease in individuals with T1DM remains uncertain. In this study, a beagle model was established to simulate T1DM, enabling investigation into the role of NAC in liver disease using RNA-seq biogenic analysis and subsequent validation through molecular biological methods. The findings revealed suppressed expression of *CXCL12* chemokine in the livers of individuals with T1DM, while treatment with NAC induced specific activation of *CXCL12* within the liver affected by T1DM. These results suggest that *CXCL12* may serve as a regulatory factor involved in the therapeutic effects of NAC on liver disease associated with TIDM. This discovery holds significant implications for utilizing NAC as an adjunctive therapy for managing complicated liver diseases accompanying type 1 diabetes mellitus.

## 1. Introduction

Type 1 diabetes mellitus (T1DM) is an immune-mediated autoimmune metabolic form of diabetes, primarily characterized by insulin deficiency and hyperglycemia. Recent research has elucidated the intricate nature of this disease, recognizing it as a consequence of complex interactions among environmental factors, microbiota, genomics, metabolism, and the immune system [1]. Although commonly associated with adolescence onset, T1DM also affects up to 50% of adult patients; approximately 50% are initially misdiagnosed as having T2DM [2]. The global incidence rate of T1DM is steadily increasing and was estimated to be around 8.7 million individuals worldwide in 2020, including approximately 1.5 million individuals under the age of 20 [3].

Diabetes can lead to a range of complications, including diabetic nephropathy, cardiovascular disease, and neuropathy. However, there is limited research on liver-related complications in relation to T1DM. Clinical observations have suggested a correlation between elevated transaminase levels and non-alcoholic fatty liver disease (NAFLD) in T1DM patients; however, conclusive evidence of a causal relationship between T1DM and NAFLD is lacking [4]. Nevertheless, certain chronic liver diseases increase the risk of developing type 2 diabetes [5]. The incidence rate of NAFLD in type 2 diabetes patients (76%) exceeds that in type 1 diabetes patients (20%) [6]. The most prevalent uncontrolled form is primary ketotic energy deficiency caused by T1DM [7]. Analysis of liver stiffness indicates severe or advanced fibrosis in T1DM [8], and clinical cases may also present with hepatomegaly [9]. Furthermore, findings from a study using a mouse model suggest that T1DM induces hepatocyte apoptosis [10]. The precise pathogenic mechanisms underlying the development of liver disease in individuals with T1DM remain elusive, despite extensive research on complications such as nephropathy, neuropathy, and cardiovascular diseases in T1DM. These investigations have encompassed various aspects including iron dysregulation, cellular apoptosis and necrosis, lipid metabolism disturbances, and oxidative stress.

Initially included in the World Health Organization’s list of essential medicines as a mucolytic agent, NAC has gradually gained widespread utilization as an antioxidant due to its exceptional antioxidant properties. NAC can be administered intravenously or orally for the treatment of severe liver damage caused by acetaminophen poisoning [11,12]. As a membrane-permeable precursor of cysteine, NAC possesses both scavenging abilities against reactive oxygen species (ROS) and effective characteristics as an antioxidant [13]. Additionally, NAC serves as a necessary precursor for glutathione (GSH) synthesis and can be used as a supplement to replenish GSH levels [14]. Apart from combating oxidative stress, NAC exhibits therapeutic potential in preventing liver diseases associated with decreased GSH levels and/or increased oxidative stress, including other pathophysiological processes such as mitochondrial dysfunction and cell apoptosis [15]. Previous studies have demonstrated that NAC exerts positive effects on various psychiatric disorders by regulating neurotransmitters, stabilizing oxidative status, and controlling inflammatory mediators [16,17]. The potent chelating activity of NAC is also harnessed for hepatoprotection and nephroprotection against heavy metal poisoning [18]. According to the findings from a clinical trial, NAC supplementation has demonstrated efficacy in enhancing management of oxidative stress, mitigating mitochondrial dysfunction, attenuating inflammatory response, improving insulin sensitivity, restoring endothelial function, and reducing genetic toxicity [19]. NAC alleviates oxidative stress and apoptosis and prevents skeletal muscle atrophy in T1DM [20]. Additionally, NAC is believed to enhance cognitive function in individuals with type 2 diabetes [21]. Subsequent to NAC treatment, cerebral vascular and blood flow abnormalities are restored in mice lacking insulin receptors on astrocytes [22]. Local administration of NAC exhibits therapeutic effects on ocular health by dissolving mucus, clearing hydroxyl radicals, and regulating inflammatory cascade reactions [23]. The robust antioxidant activity of NAC and its role in modulating inflammation suggest potential for the prevention and management of diabetes.

In our previous laboratory investigations, we have observed that the combination therapy of NAC and insulin exhibits superior efficacy in managing various complications associated with T1DM, including cardiomyopathy, hepatic disorders, and renal dysfunction when compared to insulin monotherapy [24,25,26]. However, there is a paucity of research exploring the use of NAC as an independent treatment for T1DM-related liver disease, and the underlying mechanism remains elusive. Therefore, this study aims to utilize a T1DM beagle model and employ an RNA-seq transcriptomics approach to analyze and validate the role of NAC in addressing liver issues associated with T1DM.

## 2. Materials and Methods

### 2.1. Establishment of the Beagle Dog T1DM Model

Beagle dogs, aged approximately 1 year and weighing between 8 and 10 kg, were selected as the research subjects. They were randomly allocated into three groups: control group (CON), type 1 diabetes mellitus group (T1DM), and diabetes + NAC treatment group (NAC), with three dogs in each group. The T1DM beagle dog model was established using a combination of streptozotocin (20 mg/kg) (Yuanye, Shanghai, China) and Alloxan (20 mg/kg) (Yuanye, Shanghai, China) [26,27], followed by measurement of blood glucose and insulin levels after 7 days to confirm successful establishment of TIDM. Subsequently, the CON and TIDM groups received standard feeding, while the NAC group underwent oral administration of NAC based on body weight (30 mg/kg) [28]. Clinical biochemical tests were conducted over a duration of 120 days [27,28], during which animal weight, blood glucose levels, urine sugar levels, and a 24 h blood glucose curve were recorded. Liver samples were collected post-euthanasia for analysis. All animal experimental operations in this study were approved and supervised by the Ethics Committee for Animal Experiments of South China Agricultural University (No. 2023F212).

### 2.2. MRNA Transcriptome Sequencing Analysis and Acquisition of Differentially Expressed Genes (DEGs)

In summary, clean mRNA data were obtained from CON, T1DM, and NAC liver samples through the execution of library construction, library quality assessment, and Illumina platform sequencing. Ultimately, the fragments per kilobase of transcript per million fragments mapped (FPKM) was acquired as a quantitative indicator for assessing the expression level of transcripts or genes. We carried out differential analysis using limma (version 3.40.6) [29] to identify DEGs between comparison and control groups.

### 2.3. Weighted Gene Co-Expression Network Analysis (WGCNA) and Identification of Hub Modules

The mRNA expression matrices of each group were subjected to WGCNA in order to characterize the correlation patterns between different samples and identify potential biomarker genes or therapeutic targets based on the coherence of gene sets and their associations with phenotypes. Initially, interactions between genes were analyzed using the Pearson correlation coefficient and average linkage method. Subsequently, a weighted adjacency matrix was constructed by applying a power function with parameter β as a soft thresholding parameter to emphasize strong correlations while penalizing weak ones. The adjacency matrix was then transformed into a topological overlap matrix (TOM), which quantified the network connectivity of each gene by summing its adjacencies with all other genes and calculating the corresponding dissimilarity (1-TOM). A TOM-based average linkage hierarchical clustering method was employed to analyze clusters with a minimum module size of 30 while maintaining the sensitivity level at 3. Furthermore, modules were further scrutinized by computing similarity among characteristic genes within each module and determining appropriate cut lines for merging certain modules. Finally, hub modules and module-hub genes were identified.

### 2.4. Acquisition of Intersection Genes and Hub Genes

Immunorelated gene sets were extracted from the MSIgDB database (https://www.gsea-msigdb.org/gsea/msigdb (accessed on 1 June 2024)) [30] and denoted as immGenes after eliminating duplicate values. Target genes associated with T1DM were searched in the Genecards (https://www.genecards.org/ (accessed on 1 June 2024)) [31] and OMIM (https://omim.org/ (accessed on 1 June 2024)) [32] databases using the keyword ‘type 1 diabetes mellitus’. The identified target genes from both databases were merged to establish a comprehensive set of T1DM target genes. Venny [33] was employed for intersection filtering among T1DM, module-hub genes, DEGs, and immGenes to identify common genes.

The process of feature selection involves performing linear regression using the least absolute shrinkage and selection operator (LASSO) with L1 regularization to induce sparsity in the learned feature weights, as previously described [34]. Subsequently, the identified intersection genes are further scrutinized and designated as hub genes. Finally, the reliability of the prognostic model is assessed through receiver operating characteristic (ROC) curve analysis.

### 2.5. Functional Enrichment Analysis

This study employed the Kyoto Encyclopedia of Genes and Genomes (KEGG) REST API (https://www.kegg.jp/kegg/rest/keggapi.html (accessed on 1 June 2024)) for gene set functional enrichment analysis, thereby acquiring up-to-date KEGG pathway gene annotations as a background database to identify the target gene set. Specifically, we used the clusterProfiler package (version 3.14.3) in R software to conduct the enrichment analysis and derive the gene set enrichment results. To ensure statistical rigor, each gene set was required to contain between 5 and 5000 genes, with a *p*-value threshold of <0.05 and a false discovery rate (FDR) threshold of <0.25.

For GO-based functional enrichment analysis, we utilized Gene Ontology (GO) annotations from the org.Hs.eg.db package (version 3.1.0) in R software as the background database to map the target genes to the reference set. The clusterProfiler package (version 3.14.3) was again employed to perform the enrichment analysis, adhering to the same criteria for gene set size, *p*-value, and FDR thresholds to ensure the statistical significance of the results.

Gene set variation analysis (GSVA) was performed using the GSVA R package (version 1.40.1). To calculate the enrichment score for each sample within the gene sets, we followed the methodology described by Hanzelmann et al. [35] and used the c5.Go.V7.4.Symbols GMT set from the Molecular Signatures Database (MSigDB) [36] as our predefined gene set. The minimum gene set size was set to 5, while the maximum was limited to 5000, ensuring a comprehensive evaluation of relevant pathways and molecular mechanisms. Subsequently, we computed the enrichment scores for each sample in each gene set, resulting in an enrichment score matrix. To evaluate the statistical significance of gene expression differences between the control and experimental groups, we applied the *t*-test function in the R software 4.4.0 package for two-sample *t*-tests. We then adjusted the *p*-values using the p.adjust function to control for the false discovery rate (FDR), thereby generating comprehensive differential expression data for all genes.

### 2.6. Western Blotting

The tissue homogenates were treated with radioimmune precipitation assay (RIPA) buffer, and the protein concentrations among groups were standardized based on total protein content after centrifugation. Subsequently, an equal amount of proteins was loaded onto SDS-PAGE for electrophoresis and subsequently transferred to PVDF membranes. Following that, the membranes were blocked with 5% nonfat milk for one hour. Finally, specific primary antibodies against CXCL12 (Abmart, Shanghai, China) were utilized for overnight immunoblotting at 4 °C. The membrane was subsequently treated with a horseradish-peroxidase-conjugated secondary antibody, followed by analysis of Western blot bands using Image J (https://imagej.net/ij/).

### 2.7. RNA Extraction and qPCR Assay

Total RNA was extracted using the Trizol assay, followed by reverse transcription into cDNA using a one-step reagent (Vazyme, Nanjing, China). Subsequently, fluorescence quantitative analysis was performed using the ABclonal qRT-PCR kit (ABclonal, Wuhan, China).

### 2.8. HE Stain

Following baking at 55 °C for 1 h, the tissue sections underwent deparaffinization and subsequent PBS washing. Subsequently, eosin staining and hematoxylin staining were conducted. After thorough rinsing, the sections were dehydrated using a gradient of alcohol and xylene, followed by sealing with resin and microscopic imaging.

### 2.9. Transmission Electron Microscopy (TEM)

Liver tissue samples were fixed in 2.5% glutaraldehyde (pH = 7.4, 4 °C) for a duration of 24 h, followed by overnight immersion in phosphate-buffered saline at a temperature of 4 °C. Subsequently, the samples underwent treatment with a fixative containing 1% samarium tetroxide, dehydration using ethanol, and final embedding in epoxy resin before examination via TEM utilizing a Hitachi H-7500 instrument from Japan.

### 2.10. Detection of Hydrogen Peroxide (H_2_O_2_) and Malondialdehyde (MDA) Content

Hydrogen peroxide (SAB, Sioux Falls, ND, USA) and MDA detection kits (Beyotime, Shanghai, China) were employed to evaluate the respective parameters in each experimental group. Specifically, hydrogen peroxide concentrations in liver tissue homogenates were determined by reacting H_2_O_2_ with titanium sulfate, followed by colorimetric analysis at 415 nm according to the manufacturer’s protocol. For MDA measurement, liver tissue homogenate supernatant was prepared through a process of heating for 15 min, subsequent cooling, and centrifugation at 1000× *g* for 10 min. The absorbance of the resultant solution was measured at 532 nm.

### 2.11. Immunofluorescence Assay

The liver tissue sections were fixed in paraffin, dehydrated at 55 °C, washed with PBS, subjected to antigen retrieval using citric acid and sodium citrate in a microwave oven, permeabilized with 1% triton X-100, rinsed thoroughly, and blocked with 10% horse serum at 37 °C for one hour. Subsequently, the sections were incubated overnight at 4 °C with the primary antibody and then labeled with the corresponding fluorescent secondary antibody on the following day. Finally, DAPI-sealed slides were captured using fluorescence microscopy.

### 2.12. Immunohistochemistry

The paraffin-embedded liver tissue was sectioned and subjected to immunohistochemical staining for CXCL12. The conjugated antibodies were allowed to react overnight at 4 °C, followed by incubation with the corresponding secondary antibodies for 1 h on the subsequent day. Color development was achieved using DAB, while hematoxylin was used for nuclear counterstaining. Hydrochloric acid was employed for color separation, ammonia water reversed the blue color, and then a thorough washing with running water for 15 min ensued. Finally, after undergoing a gradient dehydration procedure, the resin plates were sealed and photographed under a microscope.

### 2.13. Statistical Analysis

The data are presented as the mean ± standard deviation (SD) from a minimum of three independent experiments and were analyzed statistically using GraphPad Prism 9 (GraphPad, San Diego, CA, USA). Normality was assessed using the Shapiro–Wilk test, and homogeneity of variance was evaluated via Levene’s test. A *p*-value greater than 0.05 in the Shapiro–Wilk test indicated normal distribution, while a *p*-value exceeding 0.05 in Levene’s test confirmed homogeneity of variance. Intergroup comparisons were performed using *t*-tests or one-way/two-way ANOVA as appropriate. Statistical significance is indicated as follows: * *p* < 0.05, ** *p* < 0.01, *** *p* < 0.005, **** *p* < 0.001.

## 3. Results

### 3.1. NAC Can Regulate T1DM Liver Disease and Reduce Liver Damage

**Figure 1:**
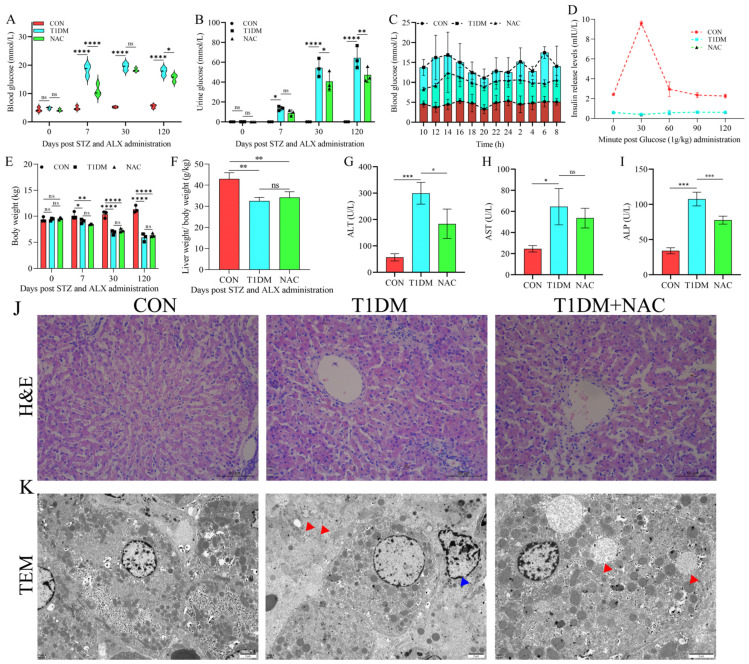
Clinical and microscopic features of T1DM liver disease. (**A**) Continuous monitoring of fasting blood glucose. (**B**) Continuous monitoring of urine glucose. (**C**) Continuous 24 h monitoring of blood glucose. (**D**) Insulin release assay. (**E**) Continuous monitoring of body weight. (**F**) Specific gravity of liver. Clinical biochemical levels of ALT (**G**), AST (**H**), and ALP (**I**). (**J**) Alteration of overall liver morphology. (**K**) Ultrastructural features of liver. The red arrow indicates the presence of autophagy, while the blue arrow represents apoptosis. * represents *p* < 0.05, ** represents *p* < 0.01, *** represents *p* < 0.005, **** represents *p* < 0.001. ns represents no significate changes. Scale bar: 50 μm for HE stain, 2 μm for TEM.

We observed that both the T1DM and NAC groups exhibited fasting blood glucose levels > 7.0 mmol/L on day 7 after modeling. Stable hyperglycemia (fasting blood glucose > 7.0 mmol/L) was also evident on day 30 and day 120, confirming the successful establishment of the T1DM beagle model (Figure 1A). Simultaneously, urine glucose levels were measured, revealing significantly elevated urine glucose in both the T1DM group and NAC group at days 7, 30, and 120 (Figure 1B). Interestingly, NAC demonstrated a significant reduction in blood glucose and urine sugar; however, it did not lower blood glucose below the threshold of <7.0 mol/L. Furthermore, continuous monitoring of blood glucose over a twenty-four-hour period showed that although NAC effectively reduced blood glucose levels, it failed to restore them within the normal range (Figure 1C). The insulin release test demonstrated that dogs in the T1DM and NAC groups exhibited low insulin levels, indicating an impaired ability to respond to changes in blood glucose levels, consistent with the criteria for T1DM (Figure 1D). Additionally, we assessed body weight (Figure 1E) and liver specific gravity (Figure 1F), revealing a significant reduction in both parameters within the T1DM group, which was not reversed by NAC treatment. Furthermore, biochemical indices such as ALT (Figure 1G), AST (Figure 1H), and ALP (Figure 1I) indicated hepatic damage caused by TIDM. To investigate the impact of NAC on antioxidant levels in T1DM-induced liver disease, we measured H_2_O_2_ and MDA levels. The results indicated that NAC significantly enhanced antioxidant capacity, suggesting its potential to mitigate T1DM-induced liver damage by improving antioxidant activity (Figures S1 and S2). However, this detrimental effect was mitigated by NAC administration. Histological examination using HE staining revealed that the CON group displayed well-organized strand-like structures with neatly arranged cells (Figure 1J). In contrast, the TIDM group exhibited disordered cells with disrupted strand-like structures and cellular swelling (Figure 1J). The NAC group still maintained visible cable-like structures; however, intercellular space enlargement and slight cell enlargement were observed (Figure 1J). Compared to the microstructure of the CON group displaying well-organized cable-like structures with neatly arranged cells, autophagy (red arrow) and apoptosis (blue arrow) were evident in the microstructure of the TIDM group while autophagy (red arrow) was also observed in the NAC-treated group (Figure 1K).

### 3.2. DEGs and Functional Enrichment Analysis in Liver Disease of T1DM

**Figure 2:**
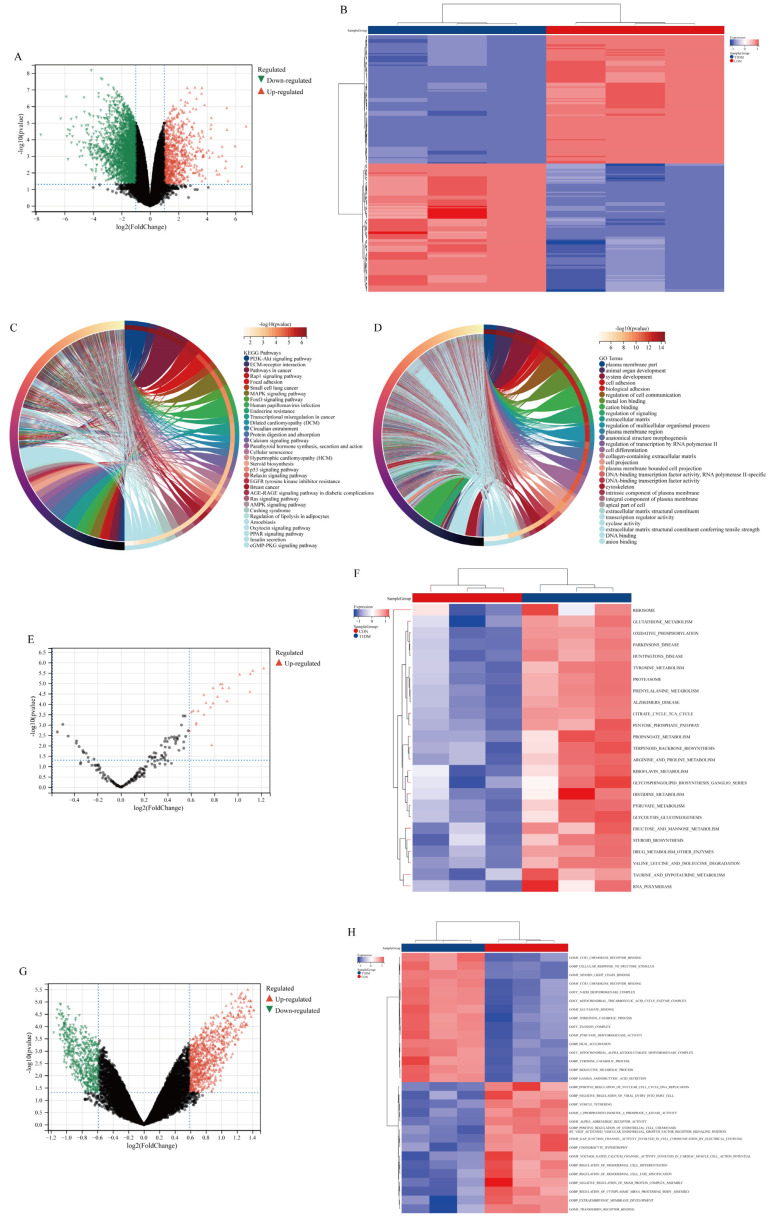
Acquisition and functional enrichment analysis of differentially expressed genes (DEGs) in T1DM liver disease. (**A**) Volcanic maps of DEGs. (**B**) Heat maps of DEGs. (**C**) Top 30 KEGG pathways. (**D**) Top 30 GO pathways. (**E**,**F**) GSVA analysis based on KEGG gene sets. (**G**,**H**) GSVA analysis based on GO gene sets.

A total of 3642 DEGs were identified, comprising 760 up-regulated and 2882 down-regulated genes. DEGs were visually depicted in the volcano plot (Figure 2A) and heat map (Figure 2B). KEGG (Figure 2C) and GO analysis (Figure 2D) revealed significant enrichment of DEGs in signaling pathways associated with diabetes, including PI3K-Akt, AMPK, FoxO, and AGE-RAGE pathways. Furthermore, GSVA analysis demonstrated similar substantial enrichment of DEGs in diabetes-related signaling pathways such as glutathione metabolism, oxidative phosphorylation, and tyrosine metabolism (Figure 2E–H).

### 3.3. Construction of the WGCNA Network

**Figure 3:**
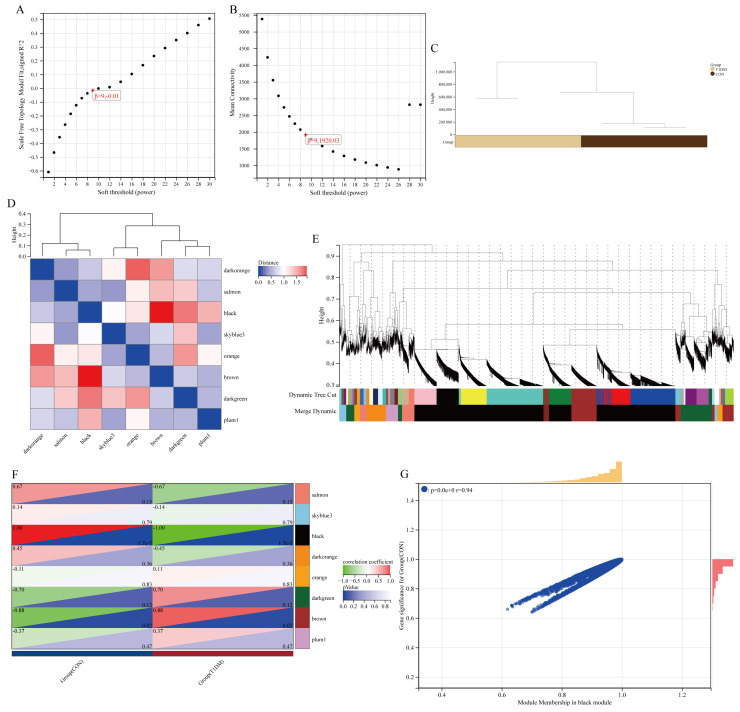
Construction of expression network in T1DM liver disease. (**A**,**B**) The fitting index for the scale-free network topology is obtained through power analysis of soft thresholding. (**C**) Sample clustering. (**D**) The heat map illustrates the topological overlap matrix of genes chosen for weighted coexpression network analysis. (**E**) The identification of coexpression clusters and their corresponding color assignments is achieved through hierarchical cluster analysis. Each color represents a distinct module within the gene co-expression network that has been constructed using WGCNA. (**F**) The relationship between the groups and 8 modules. (**G**) The scatter plot demonstrates the correlation between MM and GS within the purple module.

In accordance with the principles of scale independence (Figure 3A) and average connectivity (Figure 3B), we established the threshold for soft power. Furthermore, based on the clustering patterns of gene expression (Figure 3C), co-expression modules exhibiting a distance of less than 0.25 were merged into eight distinct groups. Subsequent analyses were performed through module eigengene clustering (Figure 3D) and gene clustering analysis (Figure 3E), which revealed a high degree of independence in gene expression across different modules. By investigating significant modules in T1DM liver disease, our objective was to identify the most substantial association level related to T2DN (Figure 3F). The findings indicated that the black module demonstrated the strongest correlation with T1DM liver disease (Figure 3F). Additionally, a notable correlation was observed between the average value of the black module and gene significance, suggesting its potential involvement in disease progression or treatment response (Figure 3G). The membership degree for each module was determined by calculating correlations between module eigengenes and gene expression levels. Applying a cutoff criterion of |MM| > 0.8, we identified a total of 4495 hub genes that are clinically relevant and tightly interconnected, referred to as ‘module-hub genes’.

### 3.4. Acquisition and Functional Analysis of Potential Hub Genes

**Figure 4:**
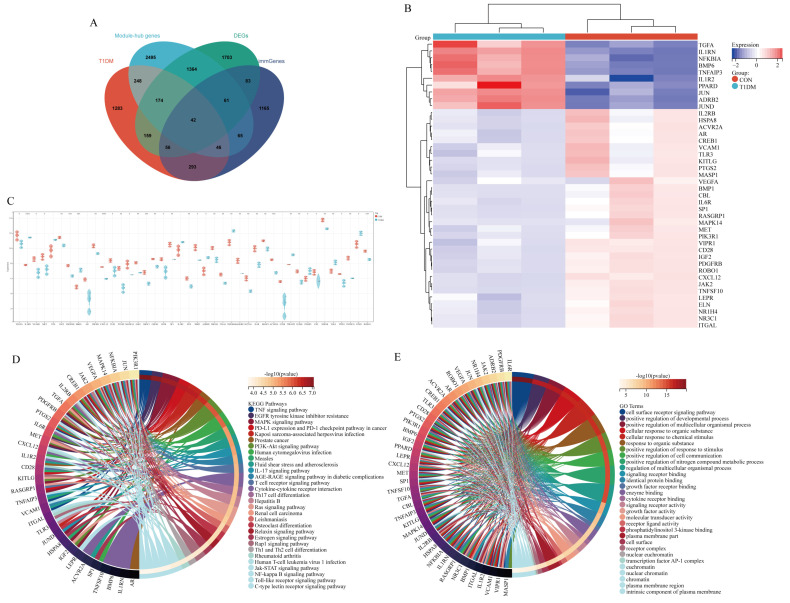
Acquisition of potential hub genes. (**A**) Intersection of immGenes, T1DM-related genes, DEGs, and black module-hub genes. (**B**) Heatmap illustrates the spatial distribution pattern of hub genes. (**C**) Expression levels exhibited by the hub gene were assessed across the T1DM and CON groups. (**D**) Top 30 KEGG pathways of potential hub genes. (**E**) Top 30 GO terms of potential hub genes.

The functional enrichment analysis revealed a significant enrichment of DEGs in signaling pathways associated with diabetes and immune responses. Therefore, to identify potential hub genes, we conducted an intersection analysis among immune-related genes, T1DM-associated genes, DEGs, and module genes. Based on the Venny, a comprehensive set of 42 potential immune-related hub genes associated with T1DM liver disease was identified (Figure 4A). The heatmap visually represents the differential expression patterns of these potential hub genes across diverse populations (Figure 4B). Furthermore, boxplot analysis effectively highlights significant disparities in gene expression between each individual potential hub gene and the CON group (Figure 4C). Subsequent KEGG and GO analyses demonstrate predominant enrichment of these potential hub genes within key diabetes-associated signal transduction pathways such as TNF signaling pathway, EGFR signaling pathway, MAPK signaling pathway, PI3K-AKT signaling pathway, IL-17 signaling pathway, and AGE-RAGE signaling pathways (Figure 4D,E).

### 3.5. Acquisition of Hub Genes

**Figure 5:**
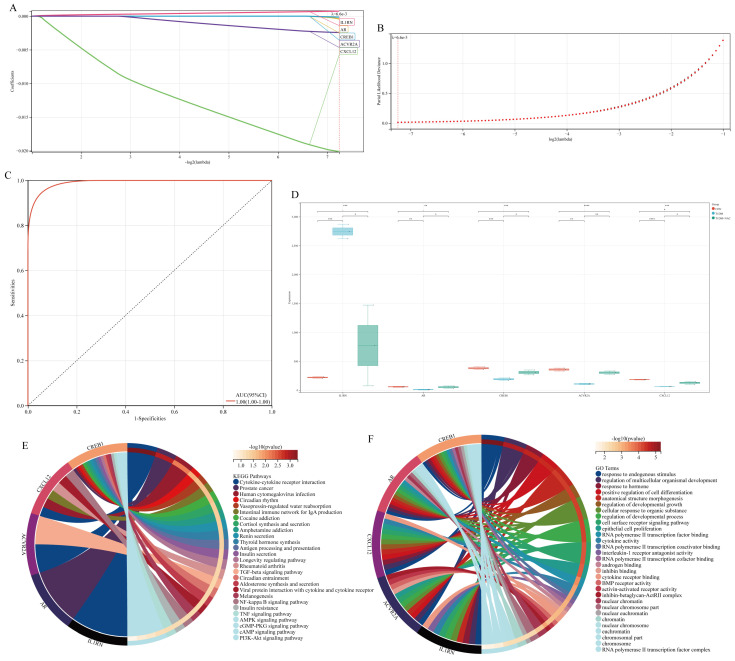
Acquisition of hub genes. (**A**,**B**) LASSO algorithm is employed to identify potential biomarkers from hub genes. (**C**) Receiver operating characteristic curve of predicted risk scores in T1DM liver disease. (**D**) Box diagram depicts the expression patterns of hub genes in each group. (**E**) Top 30 KEGG pathways of hub genes. (**F**) Top 30 GO terms of hub genes. * represents *p* < 0.05, ** represents *p* < 0.01, *** represents *p* < 0.005, **** represents *p* < 0.001.

Using the LASSO algorithm, we further identified five hub genes and biomarkers from a pool of 42 potential hub genes. We established a three-fold cross-validation framework to identify the optimal model. The Lambda value was set to 0.00659, leading to the following optimal formula: RiskScore = 0.00075 * IL1RN—0.00040 * AR—0.02012 * *CXCL12*—0.00014 * *CREB1*—0.00242 * *ACVR2A*. Ultimately, the selected hub genes included *IL1RN*, *AR*, *CREB1*, *ACVR2A*, and *CXCL12* (Figure 5A,B). The predictive performance of this model was evaluated through ROC analysis, demonstrating its high efficacy in prognosis prediction (Figure 5C). Additionally, to investigate the underlying mechanisms of NAC in treating liver disease associated with T1DM, box plots were utilized to illustrate the expression levels of these five hub genes. It was observed that *AR*, *CREB1*, *ACVR2A*, and *CXCL12* were down-regulated in T1DM-associated liver disease; however, they exhibited up-regulation after NAC treatment. Conversely, *IL1RN* showed up-regulation in T1DM-associated liver disease but decreased expression after NAC treatment (Figure 5D). Furthermore, KEGG and GO analyses revealed that these five hub genes were enriched in signaling pathways related to diabetes such as renin secretion, insulin secretion, TGF-β signaling pathway, NF-κB signaling pathway, insulin resistance, and TNF signaling pathway (Figure 5E,F).

### 3.6. NAC Reverse the Inhibition of CXCL12 Caused by T1DM

**Figure 6:**
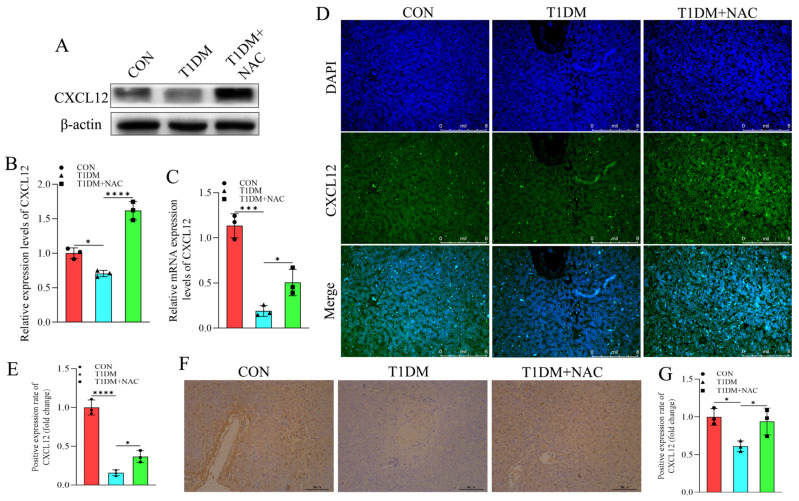
The expression level of CXCL12 in liver tissue. (**A**) The expression levels of CXCL12. Quantitative analysis of CXCL12 (**B**) protein level based on (**A**). (**C**) mRNA expression level of CXCL12. (**D**) Immunofluorescence staining of CXCL12. Quantitative analysis of CXCL12 (**E**) protein level based on (**D**). (**F**) Immunohistochemistry staining of CXCL12 (yellow) in liver tissue. Quantitative analysis of CXCL12 (**G**) protein level based on (**F**). * represents *p* < 0.05, *** represents *p* < 0.005, **** represents *p* < 0.001. Scale bar: 50 μm. Original images can be found in Supplementary Materials.

T1DM is recognized as a chronic metabolic disorder associated with autoimmunity. Through RNA-seq genomic analysis, significant alterations in the expression of CXCL12 were observed across different groups. Considering its established significance in various diseases related to T1DM, CXCL12 was selected as the subject for validation in this study. Our findings demonstrate that T1DM downregulates the expression of CXCL12, which can be effectively reversed by NAC treatment, consistent with transcriptomic analysis (Figure 6A,B) and mRNA levels (Figure 6C). Furthermore, IFA and IHC visualization of CXCL12 expression levels in liver tissues revealed a similar pattern (Figure 6E–G). Overall, our results indicate that NAC can counteract the inhibitory effect of T1DM on CXCL12, suggesting that targeting CXCL12 may play a crucial role in NAC-mediated regulation of T1DM.

## 4. Discussion

CXCL12, a chemokine that regulates directional leukocyte migration and activation in a time- and location-dependent manner during inflammation and homeostasis, belongs to the homeostatic chemokine family. This family plays a crucial role in regulating immune cell migration among lymphoid organs, blood, and peripheral tissues for immune surveillance. Moreover, chemokines are involved in various processes including angiogenesis, tumor growth and metastasis, hematopoiesis, organogenesis, cell survival, proliferation, and differentiation [37]. The pathophysiology of T1DM is characterized by the infiltration of inflammatory immune cells into pancreatic islets. This infiltration includes CD8 T cells, macrophages, CD4 T cells dendritic cells, and B cells [38,39]. These findings suggest a close association between CXCL12 and the development of T1DM with significant research value. Due to its ability to stimulate β cell regeneration, promote proliferation, and enhance survival while maintaining local immune sequestration, it has been included in research methods aimed at alleviating T1DM [40]. Furthermore, recently developed scalable in vitro differentiation protocols have enabled the production of glucose-responsive insulin-secreting beta-like cells from human embryos and induced pluripotent stem cells [41]. Therefore, whether NAC’s regulation of CXCL12 in the treatment of T1DM liver disease is linked to chemokine function remains a critical question that merits further investigation. Clarifying this relationship will enhance our understanding of the mechanisms by which NAC influences the management of T1DM liver disease and provide a robust theoretical basis for its potential clinical application.

CXCL12 is a pivotal regulatory factor in immune cell activation, trafficking, differentiation, and function. It possesses the ability to induce local immune regulation within the microenvironment of pancreatic islets [42]. Lipid nanoparticles derived from mannose modification have demonstrated their potential in enhancing skin wound healing in diabetic patients by prolonging the expression of CXCL12 [43]. CXCL12 exhibits a close association with skeletal muscle remodeling and myogenic differentiation following exercise [44]. Blocking CXCL12 can optimize the success rate and survival rate of pancreatic islet transplantation in mice [45]. Moreover, as stromal-cell-derived factor-1 (SDF-1), CXCL12 plays a crucial role in regulating immune responses, particularly in autoimmune diseases such as inflammatory bowel disease [46]. Upregulation of stromal-cell-derived factor-1α (SDF-1α) expression can stimulate endogenous progenitor cells to mobilize from the bone marrow and migrate to the site of injury [47,48]. An analysis of laboratory data from patients with type 2 diabetes mellitus (T2DM) revealed significant upregulation of CXCL12 expression [49], which contradicts the downregulation observed in T1DM beagle dogs described in this study. This discrepancy may be attributed to the normal morphology of pancreatic cells and chemotactic effect exerted by CXCL12 specifically promoting cell proliferation in T2DM. In contrast, its expression was significantly decreased in liver tissue from individuals with T1DM. Furthermore, apart from influencing anticancer drug efficacy on diabetes-associated T cells, CXCL12 also mediates firm adhesion processes for normal T cells [50].

N-acetylcysteine (NAC) modulates the tumor microenvironment by regulating the population of cancer-associated fibroblasts and inducing the activation of various cytokines, including CXCL12, SLIT2, and DCN. This modulation leads to the inhibition of tumor progression [51]. Treatment with NAC results in the upregulation of HIF-1α/CXCR4 axis via reactive oxygen species, which further facilitates NF-κB activation induced by CXCL12 and promotes epithelial–mesenchymal transition [52]. The presence of diabetes can lead to impaired wound healing capacity and compromised formation of tight junctions in both murine and human corneal epithelium. However, the application of NAC at a concentration of 100 μM has been demonstrated to ameliorate these effects [53]. NAC effectively ameliorated gestational diabetes mellitus (GDM) symptoms and the reproductive outcome of GDM mice through inhibiting oxidative stress, inflammation, and hyperlipidemia [54]. Oral NAC decreases IFN-γ production and ameliorates ischemia–reperfusion injury in steatotic livers [55]. In this study, oral administration of NAC resulted in limited bioavailability. Previous studies have highlighted the influence of the mode, route, and duration of NAC administration on its efficacy [56,57]. DPP4i has the potential to regulate tumor growth and metastasis by acting on CXCL12. In clinical verification, the anti-tumor and immunomodulatory effects of DPP4i may be offset by the carcinogenic effects of diabetes [58]. Our findings demonstrate that oral NAC administration effectively modulated changes in inflammatory chemokines associated with T1DM liver disease. However, further investigation into intravenous administration with varying concentration gradients is warranted to enhance its clinical applicability and research value.

Beagles are widely employed as test animals on an international scale, while research pertaining to diabetes in canines remains relatively limited. Given the intimate correlation between the onset, progression, management, and lifestyle of diabetes in companion dogs and their human counterparts, opting for beagle dogs as a model organism for diabetes research would undoubtedly yield enhanced clinical trial value. As a homeostatic chemokine, CXCL12 plays a regulatory role in autoimmune diseases. Impaired homeostasis causes decreased levels of CXCL12 in liver lesions associated with immune-mediated T1DM. However, NAC’s antioxidant activity restores this deficiency through ROS mediation in liver cells to exert immunoregulatory effects.

## 5. Conclusions

During the process of liver disease in T1DM, liver cells are impaired, and the expression of CXCL12 is downregulated. Meanwhile, NAC activates the expression of CXCL12 to play a role in promoting the proliferation and repair of liver cells in T1DM.

## Data Availability

The data that support the findings of this study are available on request from the corresponding author.

## Supplementary Materials

The following supporting information can be downloaded at: https://www.mdpi.com/article/10.3390/biom15020176/s1, Figure S1: The expression level of H_2_O_2_. * *p* < 0.05, **** *p* < 0.001. Figure S2: The expression level of MDA. *** *p* < 0.005.
