# Supplementary material for: CXCL12 as a Potential Hub Gene for N-Acetylcysteine Treatment of T1DM Liver Disease
Source: Biomolecules. 2025 Jan 25;15(2):176. doi: 10.3390/biom15020176 (PMC11853168; doi:10.3390/biom15020176)
Supplement: Supplementary file 1 [file biomolecules-15-00176-s001.zip › biomolecules-3384623 Supplementary material.pdf]

Supplementary figure

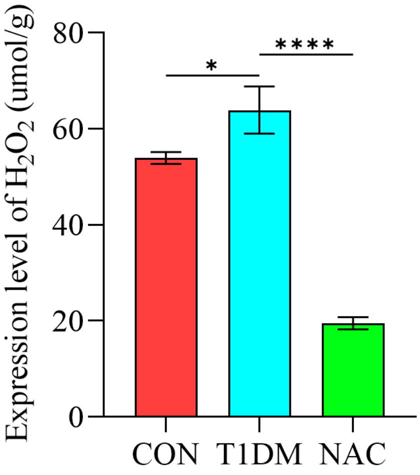

Figure S1. The expression level of H_2O_2. *$P < 0.05$, ****$P < 0.001$.

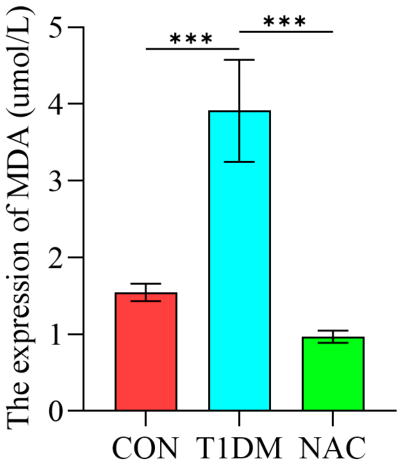

Figure S2. The expression level of MDA. ***$P < 0.005$
