# Supplementary figures and images for: CXCL12 as a Potential Hub Gene for N-Acetylcysteine Treatment of T1DM Liver Disease
Source: Biomolecules. 2025 Jan 25;15(2):176. doi: 10.3390/biom15020176 (PMC11853168; doi:10.3390/biom15020176)

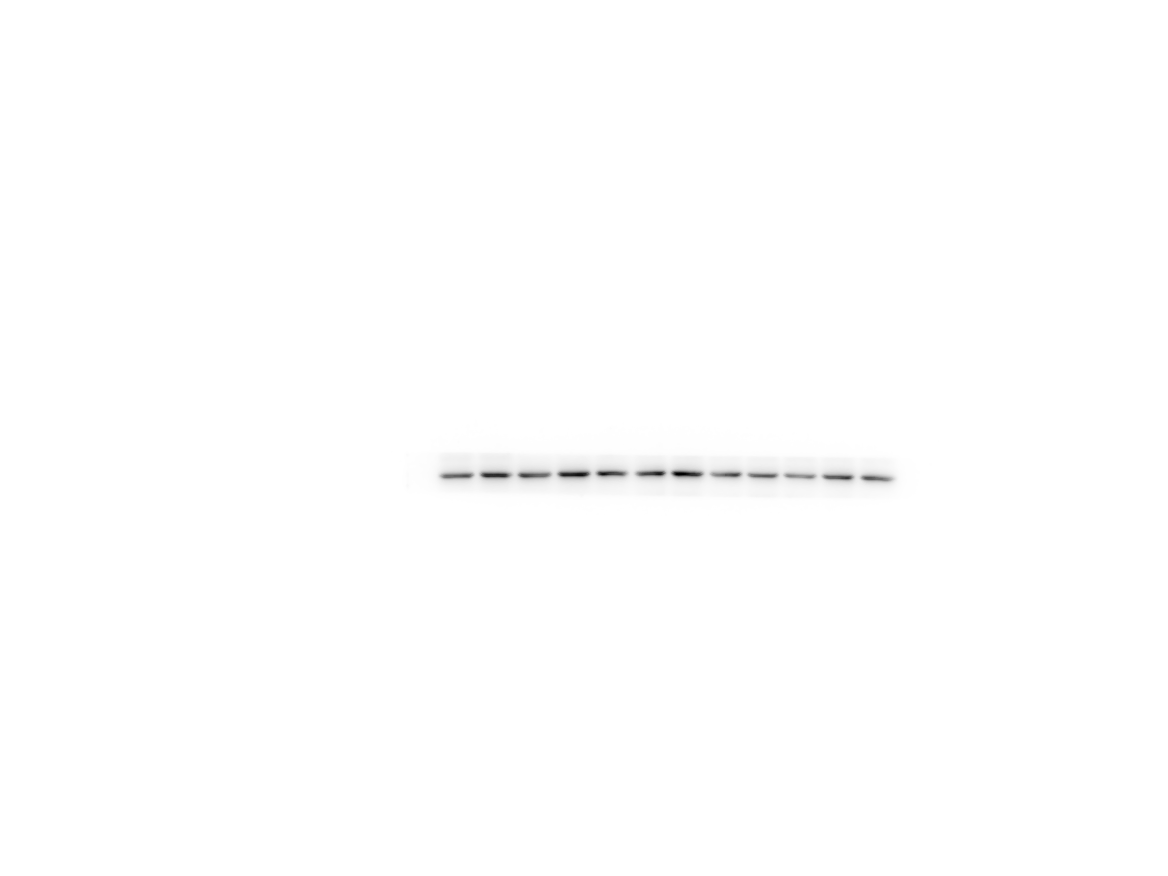

Supplement: Supplementary file 1 [file biomolecules-15-00176-s001.zip › beta-actin-1.tif]

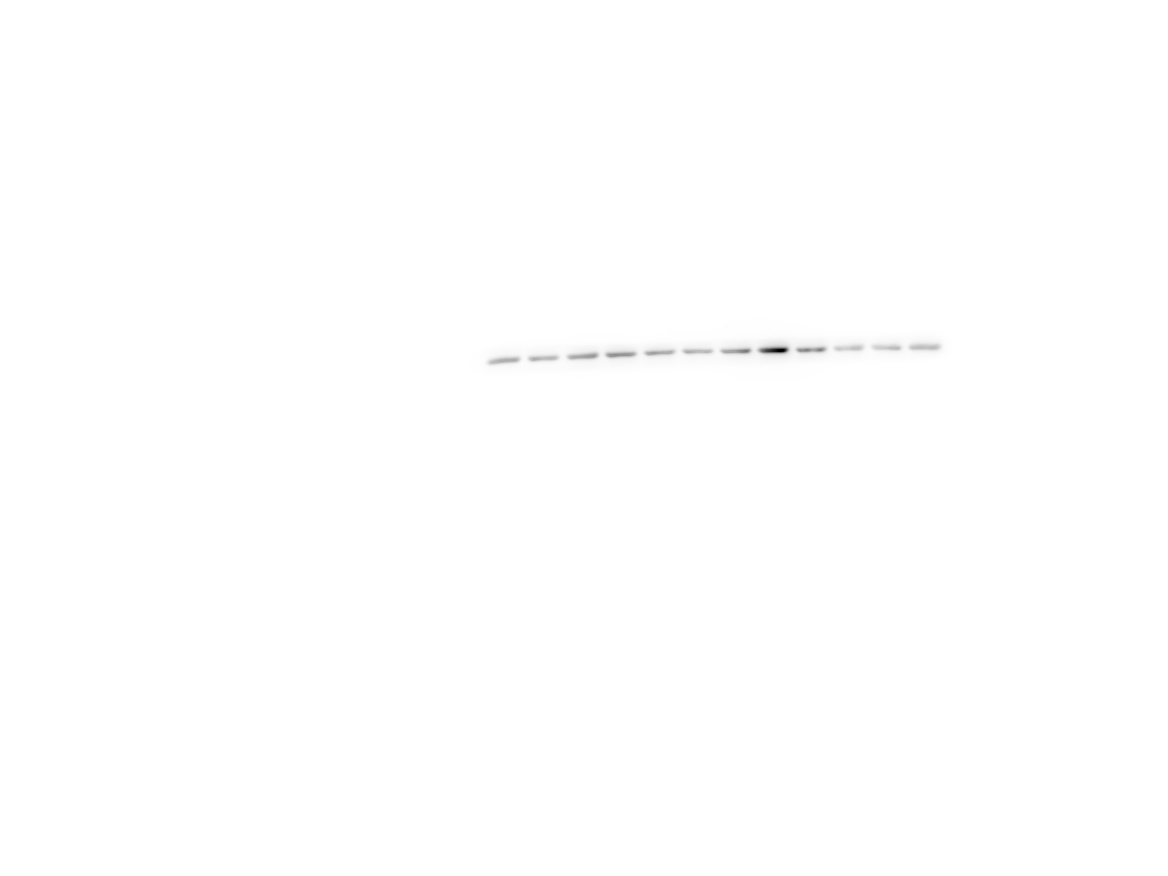

Supplement: Supplementary file 1 [file biomolecules-15-00176-s001.zip › beta-actin-2.tif]

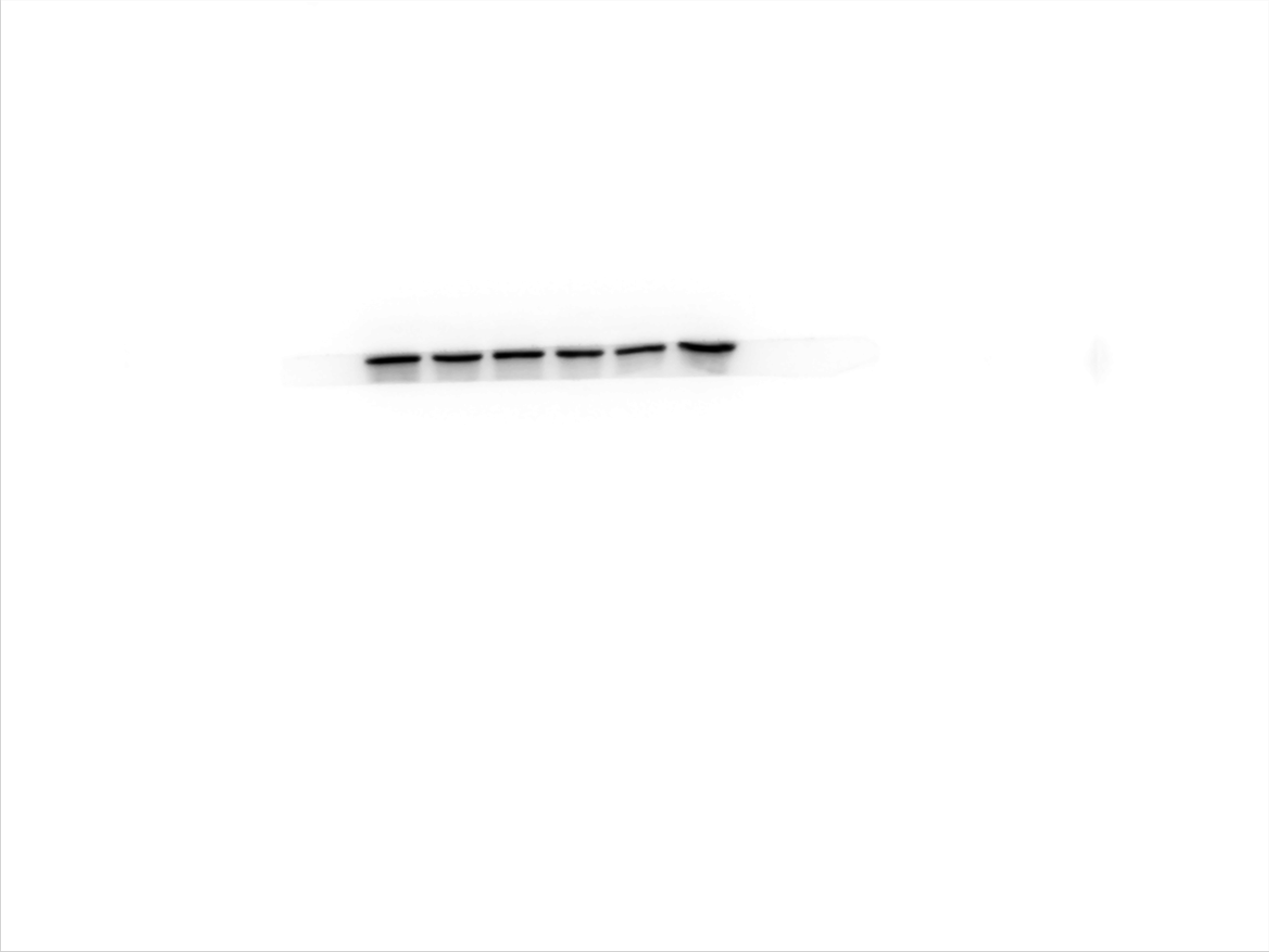

Supplement: Supplementary file 1 [file biomolecules-15-00176-s001.zip › beta-actin.tif]

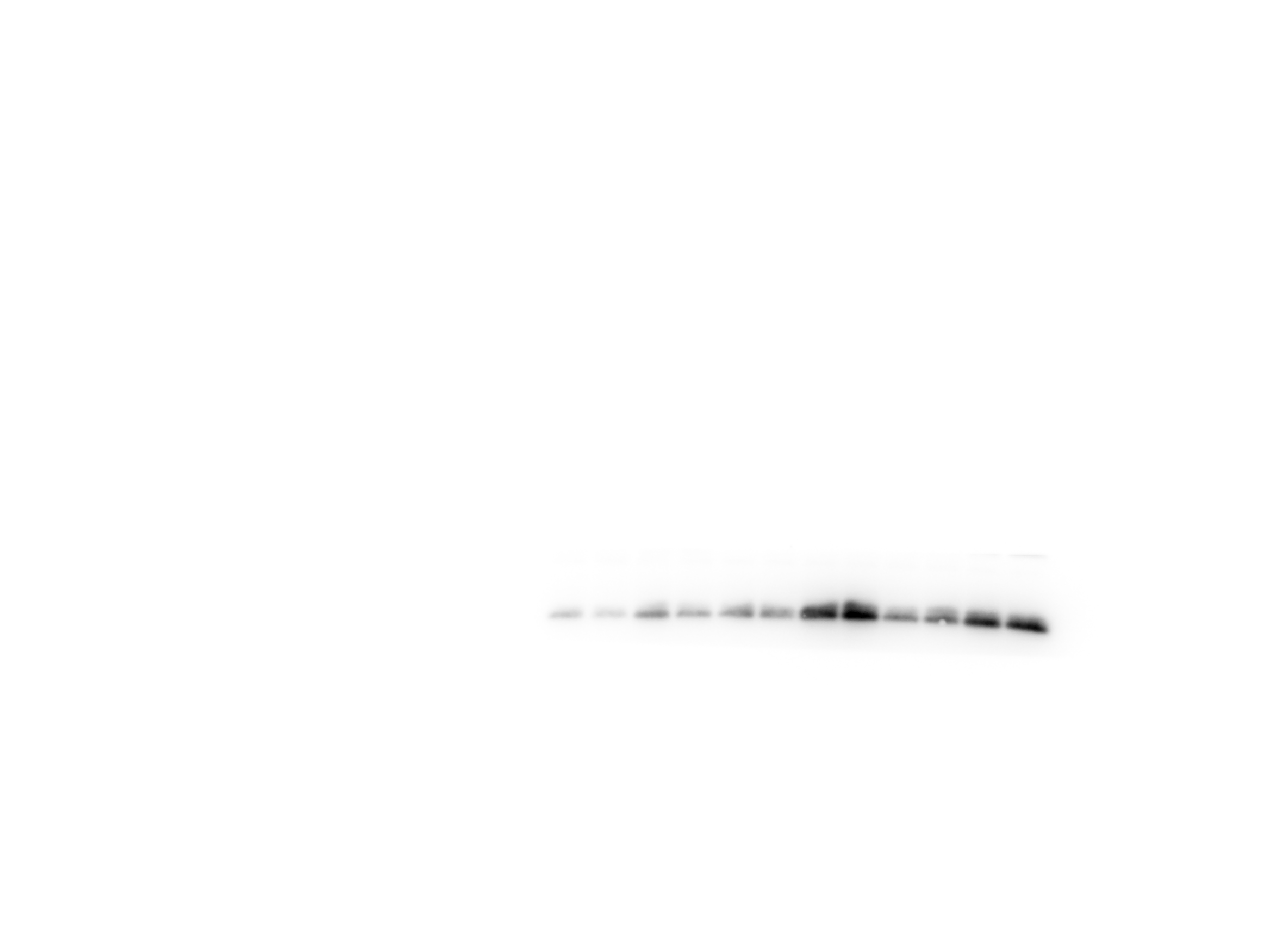

Supplement: Supplementary file 1 [file biomolecules-15-00176-s001.zip › CXCL12-1.tif]

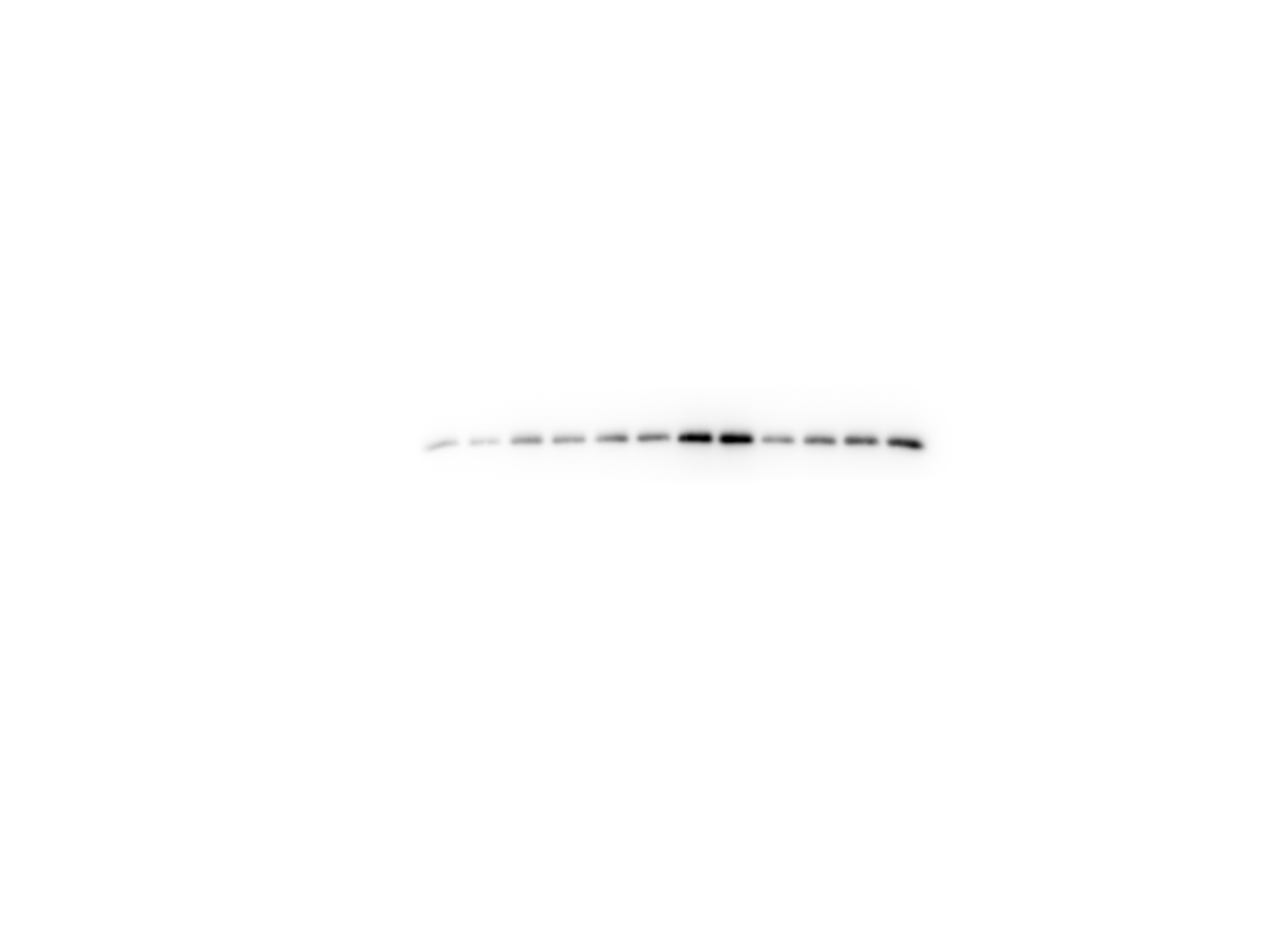

Supplement: Supplementary file 1 [file biomolecules-15-00176-s001.zip › CXCL12-2.tif]

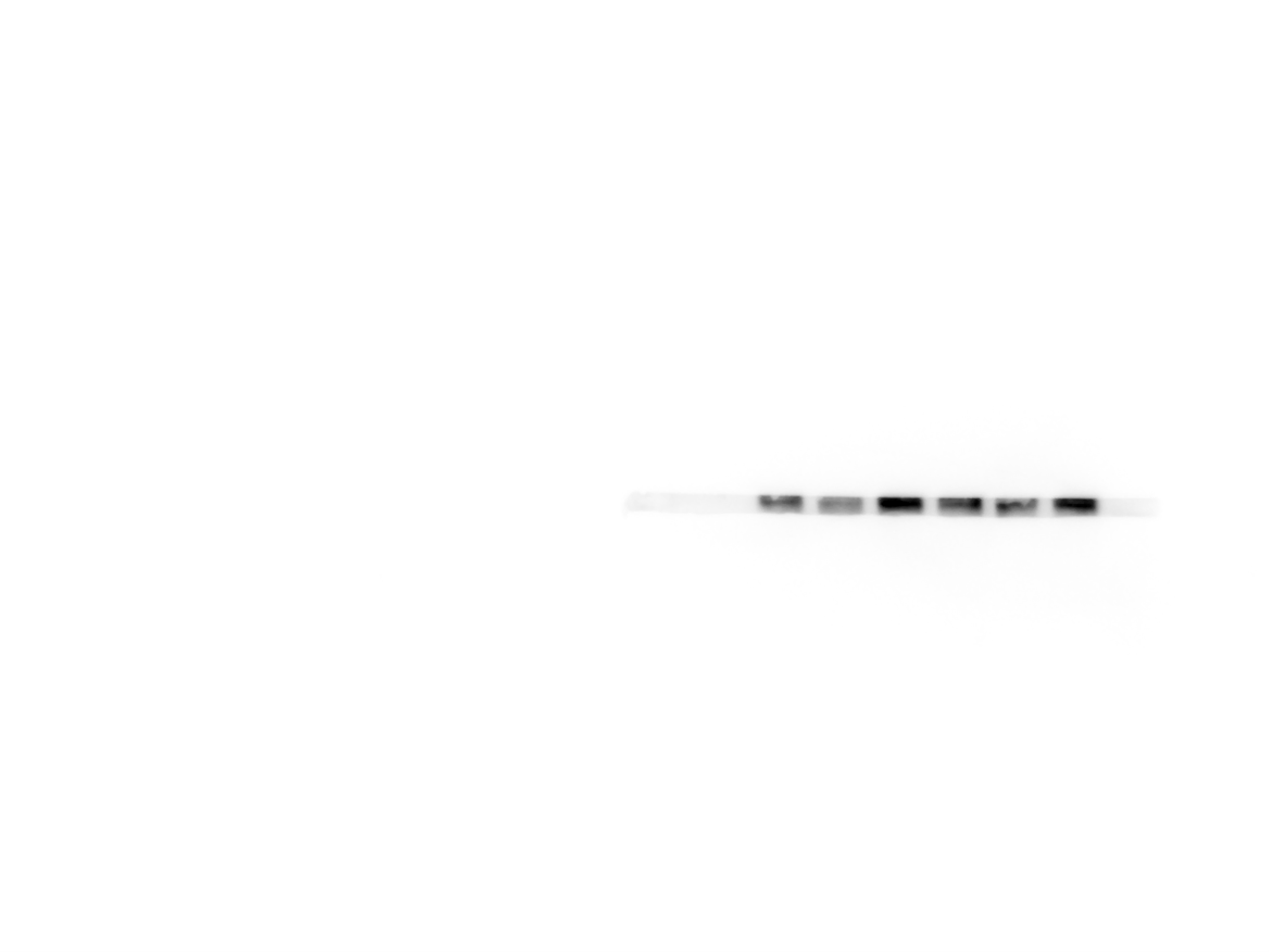

Supplement: Supplementary file 1 [file biomolecules-15-00176-s001.zip › CXCL12.tif]
